# Towards accountability-centred practices: governance in OSCEs subordinating patient and practitioner clinical experience

**DOI:** 10.1007/s10459-023-10238-7

**Published:** 2023-05-18

**Authors:** Grainne P. Kearney, Michael K. Corman, Jennifer L. Johnston, Nigel D. Hart, Gerard J. Gormley

**Affiliations:** 1 Centre for Medical Education, Queen’s University Belfast, Whitla Medical Building, 97 Lisburn Road, Belfast, BT9 7BL Northern Ireland; 2 School of Culture, Media & Society, The University of the Fraser Valley, Abbotsford, BC Canada

**Keywords:** OSCEs, Assessment, Medical education, Institutional ethnography

## Abstract

New public management ideals and standards have become increasingly adhered to in health professions education; this is particularly apparent in high-stakes assessment, as a gateway to practice. Using an Institutional Ethnographic approach, we looked at the work involved in running high-stakes Objective Structured Clinical Exams (OSCEs) throughout an academic year including use of observations, interviews and textual analysis. In our results, we describe three types of ‘work’—standardising work, defensibility work and accountability work–summarising these in the discussion as an Accountability Circuit, which shows the organising role of texts on people’s work processes. We show how this form of governance mandates a shift towards accountability-centred practices, away from practices which are person-centred; this lens on accountability-centring during high-stakes assessments invites critique of the often-unquestioned emphasis of new public management in health professions education.

## Introduction

Neoliberal ‘qualities’ have become commonplace in the delivery of modern health care, focusing on efficiency and effectiveness of care (see for example, Rankin & Campbell, 2006; Diamond, 1992; Corman, 2017). We define neoliberalism as, “the ideology that the ‘market’ and hence market-based solutions, is the most efficient and effective way to address public sector problems” (Kearney et al., 2019, p.18). McGregor (2001) explains how “neoliberal philosophy resonates with policymakers and members of the private sector,” but moreover, neoliberalism plays an important role in, “reforming health care systems … [Neoliberalism] is the common mindset in shaping health care policy reform in many nation states…” (p.82).

Central to neoliberal qualities are quantitative outcome measurements such as length of stay, response times, or waiting times, which are actively counted and used to drive policy and practice. Clarke and Newman (1997) explain this within the context of “performance management (measuring what really matters)” through a variety of quantitative technologies such as what they refer to as invisible monitoring systems geared towards “regulat[ing] labour processes” (p.62). They go on to explain:

The calculative technologies of managerialism thus provide a foundation for enacting the new logics of rationing, targeting and priority setting. Its quantitative and evaluative technologies form the basis for the new roles of contracting, audit, and regulation. (p.66).

Features of this neoliberal shift are increasingly visible in health professions education (HPE); a particular example is in relation to competency-based education where the focus on achieving competencies as outcome measures is closely intertwined with quality assurance and educational practices being organised by a strong “trust in numbers” (Porter, 1995). Connected to this discussion of neoliberalism is the related concept of new public management (NPM), which can be described as “the method by which the ideology of neoliberalism is put into practice” (Kearney et al., 2019, p.18). Whilst educators may distance themselves from active attachment to neoliberalist thought, the concepts of neoliberalism and NPM are inevitably connected to how we educate and assess health professionals as language of efficiency and accountability, for instance, are embedded within discursive devices, such as standards and frameworks, and interfacing governing and regulatory bodies that inevitably organise what counts as “competency” in HPE. Thibault (2013) explains how ensuring key competencies are achieved in HPE is carried out by “assessing whether a student has mastered them” (p.1931) through “better metrics” (p.1932). Indeed, in the context of HPE broadly, and competency-based education specifically, nowhere is NPM more evident (and evidenced) than in how competency is made visible in educational settings—through assessment. How ‘best’ to assess students is a pervasive issue with multiple assessment methods, such as OSCEs, vying to line up against assessment outcome measures.

Objective Structured Clinical Examinations (OSCEs) (Harden & Gleeson, 1979; Harden et al., 1975), at the time of their conception, provided an answer to concerns in the published medical education literature around lack of objectivity and standardisation in the assessment methods used for medical students (McGuire, 1966). Harden and colleagues claimed amongst the many benefits of OSCEs, that “In the structured clinical examination two variables, the patient and the examiner, are more controlled and a more objective assessment of the student’s clinical competence is made” (Harden et al., 1975, p.450). Now OSCEs hold a ubiquitous position in HPE worldwide, throughout the assessment of multiple professions, and through the continuum of undergraduate and postgraduate training, frequenting multiple high-stakes assessments (Norcini et al., 2018).

Whilst OSCEs as an assessment method predated the formal competency-based medical education movement, it is interesting to note inclusion of the word ‘competence’ in the above claim by Harden and colleagues. More recent definitions of assessment which are heavily referenced continue to include derivates of the word competence, a well-known example being “any purported and formal action to obtain information about the competence and performance of a candidate” (Schuwith and van der Vleuten, 2010, p.195). Whilst OSCEs are by no means the only method of assessment under the umbrella of competency-based education and assessment thereof, their repeatedly cited attributes of *objectivity* and *standardisation,* and their ability to test a particular competency in isolation, cement their place in the modern HPE assessment toolkit. In this light, much research on OSCEs focuses on perfecting their psychometric properties, which further supports and reinforces their use as valid high-stakes forms of assessment (Hodges, 2009).

In further proof of their longevity and adaptability, OSCEs similarly align well with increasing emphasis on accountability in assessment (Norcini et al., 2018). The “Consensus Framework for Good Assessment” sums up the importance that accountability plays in assessment, particularly in high-stakes assessments such as National Licencing Assessments—“the increasing diversity of candidates and programs, the importance of legal defensibility in high-stakes assessments, globalisation and the interest in portable recognition of medical training, and the interest among employers and patients in how medical education is delivered and how progression decisions are made” (Norcini et al., 2018, p.1102). Arguably the attributes in this statement also sit neatly alongside a NPM emphasis on accountability and governance in assessment. Khan et al (2013) in their AMEE guide on OSCEs linked their psychometric traits to ‘assuring quality’ in assessment.

Researchers from different parts of the world have offered a critical stance towards OSCEs. Brian Hodges (2013), a notable critic of OSCEs, employed a sociological lens to consider OSCEs, concluding that students adopt behaviours that they feel are expected of them and learn “scripts” in order to portray “competence” to the examiner. Building on the performative side of OSCEs, Gormley and colleagues (2016), wrote satirically of “ritualistic candidate performances” (p.1238) such as the students’ programmed fixed introduction or their box-ticking approach to their patient to maximise their scoring. These authors suggested such behaviours promoted “examiner-centred care,” rather than fostering the conditions where students may think of patients as individuals with experiential knowledge. They use an instance where lack of authenticity is entirely unquestioned by the student as an exemplar of *“*how we routinely sacrifice the infinite variability of human experience at the altar of psychometrics*”* (Gormley et al., 2016, p.1238). In doing so, Gormley and colleagues suggest that this push for standardisation and psychometric perfection in OSCEs could render human relationships invisible in assessment, displacing the complexities of practicing medicine in favor of a more simplistic, tick-box version.

A more recent critique of OSCEs by Bearman et al. (2021) used a socio-material lens to focus instead on the labour and context for those involved in OSCEs, describing the hidden work of constant negotiation and the multiple bureaucratic processes. They write, “Our findings illustrate the large gap between the aspirations of an assessment so fundamentally focussed on reliability and validity and the complex, continually negotiated and contextually bound practices associated with OSCE design and administration” (p.648). The work we are about to describe builds on these articles, critiquing how OSCEs have evolved to practices by which standardisation and accountability are ‘achieved’, under a NPM lens.

In this article, we take a novel approach to the much-explored tension of old in HPE assessment: that of validity. We explore the social organisation of assessment using the theory/methodology of Institutional Ethnography. More specifically, our aim is to explore how high-stakes OSCEs promote a clinical practice organised by accountability, subjugating clinician’s knowledge of the messiness of real practice and the non-standardisation of real patients. To orientate readers to this article, under ‘Methodology’ we explain this approach and why we feel it can bring a lens of critical inquiry to OSCEs, particularly exploring and explicating how neoliberal ideology is playing out in HPE. We describe our Results as three types of ‘work’—standardising work, defensibility work and accountability work; explaining how this work is sequenced and how it is organised. In the ‘Discussion,’ we explicate the outcome of such work, depicted as an *Accountability Circuit*. Crucially, we provide empirical evidence of the often alluded to unintended consequences, referred to as “hidden dangers” (Rankin & Campbell, 2006), of the training of these soon-to-be doctors, where social organisation in assessment based on neoliberal tenets shapes accountability to be the focus of and for practice. An exploration of high-stakes OSCEs, as a gateway to practice and care, provides an ‘in’ to explore the often unquestioned emphasis of NPM in health professions education.

## Methodology

### Approach to inquiry

In the section above, we described how HPE in general, and assessment in HPE in particular is becoming increasingly subject to the new public management ideals that have become established in contemporary health care itself. This shift influenced our choice of approach to this analysis, to use Institutional Ethnography. Institutional Ethnography (IE) (Smith, 1987, 1999, 2005 and 2006) is the culmination of the life work of Dorothy Smith, drawing particularly on Marx’s materialism and Garfinkel’s ethnomethodology and on her experiences in the feminist movement. In IE, knowledge is viewed as being social through and through; knowledge is socially organised by people at work (DeVault, 2008) as they interface with their work context/institutional settings and key documents or texts. As such, IE looks at what people do, their everyday activities and how these activities are organised and coordinated away from where such activities take place (Smith, 2005). It involves an iterative process of discovery, empirically studying how things work and are put together (Smith, 1987), with particular emphasis on the role texts play in people’s work. Texts in IE are “forms of words, numbers or images that exist in a materially replicable form … across time and space and among people variously situated” (Smith, 2001, p.164). Texts are often hierarchical, where higher level texts “establish the frames and concepts that control texts at lower levels” (Smith, 2005, p.212); we refer to these higher order or regulatory texts as boss texts and the lower level texts as subordinate texts. A further concept that will aid reading this article is how IE defines “work”, extending beyond work in a traditional sense of paid work to “what people do that requires some effort, that they mean to do, and that involves some acquired competence” (Smith, 1987, p.165). This includes unpaid and often hidden work, such as that of a carer or managing personal illness. This definition of work differs from the focus of the exploration of OSCEs by Bearman et al. (2021) described above; we consider the on-the-ground ‘everyday’ work of various people in OSCEs and importantly, explore how this work is organised by text-mediated social organisation. In doing so, we add to the critiques of Bearman et al. and many others of OSCEs, with particular emphasis on their dominant role in NPM infiltrated HPE.

The Institutional Ethnographic approach allows explication of the actualities for those working on-the-ground, who are trying to make their work fit into new public management style boxes. This focus on objectivity and measuring objectivity led to our choice of IE. In this study, we make visible how standardised approaches to clinical assessments result from a primary focus on accountability and this in turn subordinates patient and practitioner clinical experiences at the point of students gaining their license to work as doctors.

### The research team

In keeping with both Patton’s requirements and with IE’s recognition of the integral role that the researchers themselves play in the research (see Corman, 2021), here we describe the team involved in this research and in writing this article. This article is based on the doctoral work of GK, from which she graduated in 2020. GK is a practicing General Practitioner (GP) and at the time also a Clinical Teaching Fellow in the medical school in which the study took place. GG, NH, and JJ were all Clinical Academic GPs who also held faculty roles in this medical school at the time. All four (GK, GG, NH, and JJ) had involvement in OSCEs as station developers and assessors and GG in OSCE examiner training. MC is qualitative researcher, medical sociologist, and experienced Institutional Ethnographer who provided guidance throughout the study.

### Context for the research

This study took place in a large, undergraduate medical school in the United Kingdom (UK) under the regulatory control of the General Medical Council (GMC). Students undertook written Finals followed by Finals OSCEs halfway through their fifth and final year of medical school. Passing both these written and OSCE assessments permitted these final year students a provisional license to practice medicine in the UK and to start work as a newly qualified doctor six months later; these assessments are the gateway to becoming a doctor. This study gained ethical approval from the School of Medicine, Dentistry and Biomedical Sciences Ethics committee (Ref: 17. 29v2).

OSCEs vary from medical school to medical school. Within the research period for this study (2017–18), Finals OSCEs in this medical school consisted of 16 stations, taken over three days in blocks of five or six active stations (each with two rest stations). The first two days for all students were held in a Clinical Skills Laboratory located in a university building where the students were regularly taught throughout medical school. On the third day, the cohort of students travelled to one of five different hospital sites over the region where the medical school is based. Each station lasted eight minutes, with one minute in between for the student to leave one station and read the instructions for the next station. Each station tested one skill independent of what came before or after, generally divided between history taking, performing an examination or a procedure. Most stations involved a Simulated Participant (SP); this medical school traditionally also continued to involve some real patients in Finals OSCEs on the hospital day, which was somewhat unusual in medical schools in the UK (see Kearney et al., 2022 for more detail). Examiners scored the student against a checklist of desirable outcomes and gave a Global Score, reflecting their overall opinion of the student’s abilities. The SPs also scored each student. In total, 253 students were assessed in Finals OSCEs in this academic year.

### Data collection and analysis

In IE, research begins in the everyday actualities of its participants, often through interviews, and/or observations, specifically looking at what people do—their work broadly conceived–and how their work is organised by texts that they use. The focus then moves to “investigate how their activities are coordinated. It aims to go beyond what people know to find out how what they are doing is connected with others’ doings in ways they cannot see” (Smith, 2005, p.225). The primary researcher (GK) twice attended IE workshops given by Smith and colleagues in Toronto for training in this approach and expertise during data analysis.

In this study, the primary researcher (GK) spent an academic year (September 2017–18) observing the work of those involved in Finals OSCEs, including the Clinical Academic and administration staff who specifically worked in assessment in the medical school, in combination with students, newly qualified doctors, examiners, SPs and the external examiner, recording her observations as fieldnotes. In addition, she interviewed these listed groups, as ‘key informants’ formally and where possible during the observations, asked them more informally what they were doing and how they knew to do it. She specifically looked for texts that these various groups of people used in their everyday work, for example protocols produced within the medical school to guide the setup of OSCEs or documents from the regulatory body. Seventeen interviews took place (lasting between fifteen minutes and one hour) and 58 h of observations, including 21 h of meetings, five hours of training and 32 h of observations on the week of the Finals OSCEs themselves, which included for example times outside when the assessments were underway, observing the students as they lined up prior to starting their assessment and the examiners whilst they had coffee in their breaks. In this article, we have used the pronoun they, to prevent deductive disclosure of participants.

Data analysis was iterative, looking for and at governing or boss texts and how people used them in their work. The interview guide was designed to understand what these different people did—their work processes (concrete detail as opposed to abstract concepts)–and how they knew how to do it (what role texts played). The interview guide was developed iteratively as the primary researcher learned more about how work on-the-ground was coordinated. Observations similarly focused on what people were actually doing, as their work. The primary researcher followed threads of inquiry that she learned about in her observations, formal/informal interviews and through analysis of the texts; these threads were discussed iteratively within the wider research team with a reflexive focus on positionality throughout.

As per other IE studies published in this journal (MacKinnon et al., 2020), we present our findings here as the everyday work processes of those on-the-ground, specifically noting the governing role that texts play in these work processes. In the next section, we first describe the work of standardisation, looking at why it is problematic and yet still valued and valuable. Next, we explicate defensibility work, unpacking how work on-the-ground is made visible for governance. Finally, this is traced to work of accountability, outlining the language and texts involved and how this aligns with new public management.

## Results

### Standardising work: “we don’t believe in examiners standing out”

A dominant discourse featuring throughout the observations and interviews was how people ‘did’ standardisation, as a key tenet organising their work processes. Specifically, Clinical Academics and those in administrative roles in assessment conspicuously and frequently discussed the need for standardisation in much of their work. This was particularly notable during the huge amount of preparatory work that took place in the six months leading up to the Finals OSCEs. Here we give a small number of examples firstly in their preparatory work for the OSCEs, secondly during the week of the OSCEs, and finally in the aftermath of the OSCEs, all of which are geared towards making the OSCEs as standardised as possible.

Instances of standardisation in preparatory months were apparent during the training provided for examiners and for SPs. The examiner training was a large-scale event considered by the team as one reason why the statistical reliability of the OSCEs (as measured by Cronbach’s alpha co-efficient) was favourable. This mass training permitted the planning team to “have more control over the examiners,” as per one of the Clinical Academics. In the training, there was an emphasis on standardising how the examiners would score the candidate in the more subjective aspects of scoring (in particular the Global Score). It was explained to the examiners, both new and experienced, how the planning team had a Standard Operation Procedure (SOP), utilised when they detected “variance” in examiner making, where they could adjust “outlying” examiners’ scores when deemed necessary (for example where one examiner scores statistically differently to the others marking the same station). This process of detecting variance and adjusting accordingly, as organised by the SOPs, and overseen by the GMC, the regulatory body governing the OSCEs and clinical practice, was central to the production of ‘quality’ in the assessment process.

Similar standardising processes were present with regards to the SP training sessions. For example, in the preparatory phase, it was explained by a member of the administration staff that “unless they’re properly trained, they’re not going to do their job properly and that’s going to impact on the exam for the students”. A Clinical Academic explained the following in regard to how standardised the SPs were: “Well, I think it’s, it’s standardisation, it’s scripting so that you can actually test a particular station”. Standardisation appeared to be a hallmark of their assessment processes.

During the week of the OSCEs themselves, the students’ briefings included the expectations of their appearance by the medical school during the OSCEs for example to wear their identity badges and be “bare below the elbows” where they have no clothing or jewellery (outside a wedding band) on the bottom half of their arms, a common expectation in clinical practice. Last minute checks of the stations by the team focused primarily on making sure the stations looked identical in the different circuits which ran concurrently, down to the detail for example of the exact positioning of the student instructions on the outside of the stations. In the aftermath of the OSCEs, much work is done in the name of ‘quality assurance’ of the assessment. Psychometric measurements were used to support the claim of quality of the assessment texts as well as the work of the examiners, the SPs, and the students themselves (for example using Cronbach’s Alpha co-efficient). Any variability on the scoring by the examiners or indeed of the stations themselves was actively sought out and “corrected” using psychometric means. A Clinical Academic explained, “there are a couple of hundred examiners, you are bound to have one or two who don’t behave … as expected and they stand out from the crowd. We don’t believe in examiners standing out”. So far, we have given concrete examples of standardising work, as carried out by people. We move now to explain the language and texts involved in socially organising standardising work.

Key to IE is careful attention to language, with a particular focus on how language carries social organisation; those in charge of assessment deliberately used the words standardising and standardisation. This was also the case for the students who accepted (and even sought out) this tenet of their assessments. This language was normalised in a way that any attempt to critique these practices of standardisation or quantification were absent. With the focus in IE on how texts carry the social organisation, those responsible for planning the assessment produced a number of subordinate, ground level texts which organised the work in OSCEs, two of which are specifically discussed here. A ‘blueprint’ was used to plan the OSCEs to ensure that the range of specialties and domains such as history taking, examinations and so forth were covered, reflecting biomedical categorisation. The blueprint used was derived from a document supplied by the regulatory body. A newly qualified doctor who was involved was in planning the assessment (having themselves been assessed in this way in the previous year), described the blueprint as “the bible, the bureaucracy” suggesting it may hold governing or unquestioned power. The other example of an organising subordinate text was the marksheet. The marksheets were produced by the team who planned the OSCEs; they became the textual representation of the commitment to and production of standardisation, developed with close scrutiny of the guidance from the regulatory body. A huge amount of work went into developing these marksheets for each station—an institutional template was filled, which was then road-tested, referred to in the training of the SPs, filled in by the examiners on the day and later used to produce the psychometrics of the assessments, for the students, for the examiners and for the stations. This work of standardisation as carried out by those responsible for the assessments becomes textually represented by these marksheets. This is what the students, SPs and examiners orientate to, and what their work is organised by.

### What is problematic about prioritising standardisation?

Having described the dominant and unquestioned work of standardisation, we now discuss why this may be problematic in the development of these students. In IE terms, we question what is lost through this very objective standardising approach. In this study, students described a number of concerning effects on their development as they undertook these high-stakes assessments. Firstly, students described how they presented themselves to pass this assessment; their assumption of standardisation of the assessment material organised how they prepared for and conducted themselves on the day. Whilst a Clinical Academic described that their stated aim, when setting these assessments, was to be able to recognise students who have spent time with patients, a student however stated, “when it comes to that revision phase, there was just so, so much that it was quicker to do at home. Keep going, keep going, keep going. Whereas to see patients, you’ve obviously got to go into hospital, then you have to go to the ward, find a patient that’s suitable. Takes a lot longer maybe to do one thing”. The newly qualified doctor joked of students spending time with the friends in coffee shops as opposed to clinical environments, preparing for their assessment. Students were being driven away from the patients whom they would soon be treating in the search for or in response to the push for standardisation and standardised ways of knowing and demonstrating their ‘knowing’. Students described the strategies they developed for the day of their assessments, “You can almost like trick an OSCE, you can very much prepare for it, and you can deliver your lines”. Whilst it could be argued that any student attempting any assessment will orientate to how to pass, when the assessments in question are the last hurdle before they start work as doctors, one might hope that these medical students should be thinking about their future practice with patients.

The second concerning outcome was how students oriented to an objectification of patients. An examiner commented on how they found students’ focus to be on the marksheet as opposed to the person in front of them, “It had felt like a typical OSCE in that more of the interaction was between me as the examiner and the student rather than between the student and the patient”. An SP also reflected this, when referring to the students, “I think a lot of them … they learn this mode, they go into the checklist and forgot that they have got their patient”. Students are organised to consider the person in front of them as their means to achieve what is on the marksheet and prioritise a biomedical over an experiential approach to treat this patient as an individual.

Thirdly, students explained how they oriented to standardise/objectify themselves in the assessment. They described how they are “not themselves in OSCEs” in order to pass. An SP commented on how “robotic” they are, and an examiner agreed, “so robotic and so fake and similar and military”. Students discarded their individuality to streamline themselves in an attempt to please their examiners—their appearance, behaviour, and words–depersonalising themselves as they thought it was what was demanded by what the marksheet might reward. Their socially organised ‘fakeness’ in an OSCE allowed them to score well. An examiner summed it up when they said, “I understand that this is the only way that they can standardise things, I get that. But I don’t think it is examining *them*. I really don’t think you are getting a flavour of somebody”.

Finally, and perhaps of most concern, the drive towards standardisation displaces the art of complex clinical practice—connections between students and patients–causing students to take a backward step in their professional development. Students sacrifice relationship-forming with patients due to the constraints of time and the need to gain objective marks on a rigid, biomedically oriented marksheet. These assessments are far from clinical practice, which is dynamic and uncertain, and from real patients who are individual and complex. Examiners described how they observed students “acting empathy”, unsurprising as students orientate to the marksheet.

In summary, OSCEs attempt to standardise what cannot or perhaps, *should not*, be standardised—patients, students, and human connections between them that occurs within a particular context. This approach to assessment subsumes and subjugates relationship-forming between individual students and individual patients, just as these students are gaining their license to start to care for patients. Problematising this process, a student summarised it as follows, “So, I definitely think than an OSCE isn’t about being nice to a patient or being caring. An OSCE isn’t about being the best you or the best doctor. It’s about getting the most marks you can get and making sure that you are getting over the pass line, so you don’t have to re-sit it”.

### Why strive for standardisation then?

This debate around standardisation is not new and there continues to be much discussion of the relative merits and downfalls of clinical realism versus heavily standardised teaching environments (Gormley et al., 2016; Johnston et al., 2020). In the experience of the authors who are clinicians, students frequently ask for more standardisation of teaching, and as a result teachers strive to fulfil this demand. During this study, the Clinical Academics and the administration staff were asked directly by the lead author why they placed such an overt emphasis on standardisation in all things. These informants invariably talked about standardisation as a vehicle of fairness for the students. When asked to elaborate, a Clinical Academic described how fairness in assessment was the ability to “replicate” all aspects of the assessment; another described this fairness in more psychometric terms, “Now the OSCEs, by providing standardised material, give us a much more reliable assessment of whether our students should pass or fail. So, you lose a little bit on providing holistic and realistic, you know the validity, but you win hands down in providing a fair and reliable assessment”. What counts for this Clinical Academic is reliability that the team can prove and stand over.

A frequently discussed example of unfairness often quoted during interviews with various participants was where an SP would “deviate from the script”, say something outside what their specific biomedically-focused training had prepared them to do. Based on their experience in OSCEs, a member of the administration staff explained that when lack of standardisation was perceived by a student, the “students will feel really frustrated and it can affect their performance … they’ll maybe then get really distracted by the fact that they felt that their station was different to somebody else’s station …”.

Similarly, students explained that they valued standardisation as it assured them that the assessment would be the same for all students, also seeing it as a marker of fairness. One student referred to this assumption of standardisation in their OSCE assessments, as the “silent agreement” between the students and the medical school. This student went on to explain, “I think it’s because everything from the start you’re told OSCEs are standardised, OSCEs are fair and that’s kind of the agreement, the silent agreement”. Their medical school training organised students to expect this standardisation in assessment, seeing this as an improvement on historic models of assessment students have been told about by senior doctors. One student compared the past to the present when they said, “People have talked about back in the old days you had a where long case, and it was completely random what you got. And people will recognise that that standardisation is a good thing … I think people recognise that they are not the nicest thing in the world, but compared to the other things, they are a better evil”. As this line of inquiry around how standardisation equated to fairness developed, we began to question the unintended consequences or “hidden dangers” alluded to above in medical education, where perceived fairness delivered as standardisation of assessment has become so important. A clue to explicating this came from the external examiner. They explained in interview that in their experience, students complained if they became aware of lack of standardisation in their assessments, almost framing students as would be consumers of medical education in a commercial or managerial sense. This began to shift our thinking as to why medical schools were so keen for standardisation.

### When standardisation was considered absent

As we discussed perceived fairness in OSCEs, participants described the means for students to lodge a complaint about a part of their assessments and appeal their marking within this medical school. Whilst the terminology and surrounding procedures will vary among medical schools, the underlying principles are likely to be universal. A member of the administrative team described how procedural irregularities resulted when “students spot the absence of standardisation”, using examples stated above of SPs delivering their lines differently. To claim a procedural irregularity, a student would fill in a particular form, the details of which was then investigated, gathering information such as which circuit it was, which examiner it was and whether they attended training. This was stated as an example of the increased governance processes and SOPs now in place surrounding assessment. Over the years, staff were dealing with an increasing number of appeals, reflecting a societal change towards consumerism—the ethos that students increasingly see themselves as consumers (Naidoo and Jamieson, 2005)–whereby people were more likely to complain and appeal. So was the dominance of standardisation, rather than solely to be down to creating fair assessments, in part an attempt to reduce student complaints and appeals, which would require reporting to and investigation within the wider university. Was *defensibility* in assessment processes really underpinning the importance of standardisation?

### Defensibility work:–“making myself defensible”

On considering this work of defensibility, where standardisation of all elements of an assessment helped those on-the-ground defend their work, we looked critically at why defensibility seemed important. What work required defence and whom was this work being defended against?

### Why the need for defensibility?

The need for defensibility of work came up regularly throughout interviews and observations. A Clinical Academic explained that “defensible means that you follow a principle that doesn’t depend on the person in front of you”. In other words, you set and document processes that you then can prove that you follow, regardless of the student, examiner or SP involved. Another individual explained how they were “making myself defensible”, in that they were keeping very clear documentation of their work. Whilst the assessment team spoke of the need to be able to justify their decisions to individual students should they appeal, they also explained their altruistic motivation for the benefit of both the students and wider society, in “doing the right thing by our students” and of ensuring that only those ready to be doctors are passed, with a nod to the significant consequences of being awarded this particular degree. In IE terms, this altruistic motivation could be considered to be socially organised through their experience as clinicians and their responsibility in preparing the next generation of doctors for practice. In addition to their perceived obligations to students and their future patients, the work of those in charge of these assessments also had to be defensible, in a governing sense, to the medical school and the central university. Looking up higher in the institutional chain, their work ultimately had to be approved by the regulatory body. This defensibility work involved conducting and documenting their work in a way that they could prove met the regulations laid down by the regulatory body; this was the bottom line of these assessments. A Clinical Academic explained their opinion that “defensibility is probably a big element of what the GMC wants in assessment”, explaining that “why I mention defensible is [that in] examinations, standard setting, pass[ing,] failing, there is professional judgement on an arbitrary basis. It’s arbitrary where we set a pass mark for an exam. Now if you build in a process which uses professional judgement, to objectively arrive at that arbitrary pass mark that’s viewed … as defensible”. Through this defensibility work, it seemed that more than the performance of the students was being assessed in this assessment.

### Whose work is being assessed in assessment?

Through this ethnographic work, it became apparent that the work of many people in assessment were subject to scrutiny and measurement. Considering first the examiners, they received feedback comparing their examining to the other examiners and where their scoring is deemed to be at “variance”, this is then adjusted for statistically, according to an SOP in a way that this is clearly explained at their training. In addition, examiner conduct is also under scrutiny where they are briefed to adopt a “neutral” expression in the assessment and their scoring examined after the assessment. A further element under evaluation are the individual stations, through external examiner feedback sought in advance of the assessment as to their feasibility and clinical application and after the assessment by statistical interrogation of the metrics.

Fundamentally, the assessment process as a whole was under close inspection. The on-the-ground work of everyone in assessment was open to scrutiny, with the united mission as to ensure the same assessment for every student. This might in some way explain the levels of stress on the day; standardising what is hard, if not impossible to standardise—giving the appearance of standardisation–required much work. With this work being assessed and even measured, comes the potential for comparison amongst medical schools such as in league tables. As neoliberal reformation sweeps through higher education, there is a problematic impetrative to ‘perform’ well in league tables to attract students and boost university finances through student numbers. Potential measurement of assessment processes and outcomes supports a neoliberal push towards comparison and ranking. By at what costs?

Using the lens of IE, tracing this defensibility work back, it became increasingly clear the driving force was down to the need to be accountable. This defensibility work, as played out in the work of standardisation was under the ruling relations of accountability, where ruling relations are defined as a “complex of objectified social relations that organise and regulate our lives in contemporary society” (Smith, 1999, p.73). But who or what was this work accountable to?

### Who is this defensibility work accountable to?

This on-the-ground work, including the documentation and documenting of, ultimately was to ensure defence of the standardised assessment to the governing body—the regulatory body. This regulatory body would grant a licence to the graduates of the medical school permitting them to begin practise as doctors. It was the responsibility of the medical school to be able to evidence how their day-to-day work in assessment complied with institutional processes, developed in the offices of the regulatory body many miles away from clinical workplaces. This defensibility work was how they made their work visible to the regulatory body, how they ‘quality assured’ the work.

In this ethnographic research, this thread of focus on the need for standardisation led back to the regulatory body and just as importantly, to their published documentation, which we will now discuss. The ruling relations of accountability to the regulatory body, organised accountability work.

### Accountability work: “following the rules”

What might accountability work look like, under the governance of the regulatory body? What might count and be counted under the dominant accountability discourse? At the outset, it is important to note that the same regulatory body overseeing the assessment also governs the licencing for all practising clinicians, including those who are involved in planning and in examining the assessment. On their website, the regulatory body in this jurisdiction summarise their role as follows, “We work to protect patient safety and improve medical education and practice across the UK” (GMC website). As such, the regulatory body positions themselves as the guardian to protects patients from the deliberate and undeliberate unsafe practice of doctors. They achieve this through the need for every licenced doctor to be accountable to them whereby they have the power to investigate any allegations of wrong-doing (inside and outside practice) and even where the doctors themselves may be suffering from ill health. This power extends to medical students and indeed to their educators (themselves often licenced by the same regulatory body). The accountability for the education and assessment leading to the licencing of medical students therefore lies with the medical school and specifically with those involved in this pivotal, high-stakes assessment. This governance around patient safety above all things infiltrated the on-the-ground work in assessment. One of the Clinical Academics likened this to “following the rules”. As previously with the work of standardisation, we will now give specifics of how the texts and language carry the social organisation of accountability.

### Texts socially organising accountability

We have previously discussed some texts in the section on the work of standardisation, such as the blueprint and marksheet which while specific to this medical school, will be replicated in similar forms in any medical school running similar assessments. Here we discuss what IE refers to as ‘boss’ or ‘regulatory’ texts, as defined above, used in all UK medical schools (and replicated throughout the world) which socially organise and make accountable this assessment work.

At the time of the study, there was no specific ‘assessment manual’ from the regulatory body; the boss texts that coordinate what happen in assessment are a little more subtle than this. The participants of the study often referred loosely to “GMC documents” to justify how they knew some aspect of assessment had to happen in a particular way or what their SOPs were based on. Specific questioning about this led to the document that these sweeping statements were referring to, “Outcomes for Graduates”. This is one of a number of GMC documents which “set the standards expected of medical training organisations” (GMC, 2020). It specifically “sets out the knowledge, skills and behaviours that new UK medical graduates must be able to show” (GMC, 2020). Outcomes for Graduates came up by name at many points of data collection; for example, it is linked to directly on the online guidance provided for medical students on assessment and a picture of its cover shown as a PowerPoint slide at the examiner briefing. Could this lead to speculation that reference to such a document from the regulatory body in relations to these assessments was a veiled threat to these students and examiners, reinforcing their need to comply with the instructions?

### Language socially organising accountability

As previous stated, language in IE is not neutral, rather, how we talk, and the written text carries social organisation. Here, we look at how the language choices organise the work and perhaps the threat of accountability underlying the work, alluded to above. Whilst we previously noted how the words ‘standardising’ and ‘standardisation’ regularly came up in the everyday work in assessment by both staff and students, it is interesting that there is little specific mention of standardisation in the various GMC documents or website. There is instead mention of the related concepts “common thresholds” and “consistency”.

A further interesting use of language was where some of the archetypal phraseology of the regulatory body was used out of context, in what might be considered a foreboding way. An example of this was during the student briefing when students were reminded of the need to keep assessment material confidential; lack of doing so, they were informed, could result in the regulatory body calling into question their “fitness to practice”.

### Accountability and new public management

As discussed in the introduction, there is a body of IE research interested in ‘new public management’(NPM). This vein of research examines how changes in public sector services are being organised by private sector management discourses and methods, including health professions education broadly and medical education specifically (MacKinnon et al., 2020; Webster et al., 2015). As this research progressed, alignment to the terminology and ideology of NPM became more apparent. There are numerous examples in the language of on-the-ground accountability work, where management terminology had crept into the everyday discussions—talk of stakeholders, recruitment, blueprints, procedural irregularities, quality assurance etc. In the regulatory body texts, such terminology was widespread, particularly when referring to the increased regulation into assessment that was afoot, around Key Indicators and Quality Assurance etc. This institutional terminology that dominated the discourse around regulation had also become adopted on-the-ground; it had become the common, unquestioned, and unproblematised language of those involved in what is essentially a clinically based assessment. These high-stakes assessments align with new public management ideals as a standardised and defensible assessment to ensure competence in an efficient manner with increased regulatory emphasis on quantitatively and objectively proving ‘quality’; where accountability is core.

## Discussion

### Sequencing the work as an accountability circuit

In order to unpack how a shift towards the ideology of new public management in these high-stakes assessments might impact the practices and behaviours of these senior medical students, we undertook to map out this analytic thread as an accountability circuit (defined below). Through this circuit, we illustrate our conclusion that the dominating work processes of standardisation, defensibility, and accountability above all things, displace and distance the actualities of patients and clinical practice in high-stakes assessments such as OSCEs, demoting a person-centred ethos in favour of a shift towards *accountability-centred practices.*

### Mapping the accountability circuit

**Fig. 1:**
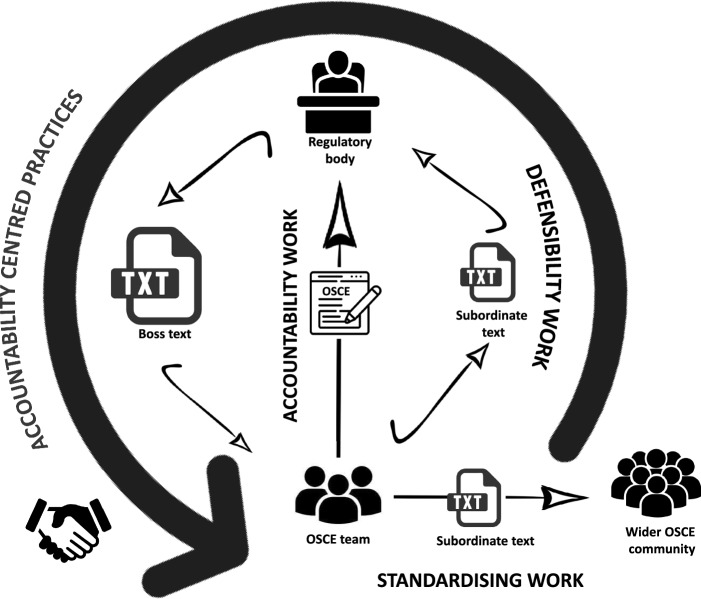
Accountability circuit; *towards accountability centred practices*

Here we present this analytic thread of accountability, accomplished through work, texts and language, mapped as what Dorothy Smith calls an accountability circuit (Griffith & Smith, 2014)–see Fig. 1. In brief, we show the work of standardisation by those involved in planning these assessments, exemplified by their production of local texts such as the marksheets, to which the student, examiner and SP orientate. We show how they translate this to defensibility work, organised (mandated) by the regulatory body through higher level or boss texts, with the collective orientation of the need for all work to be accountable. To fit within a simple diagrammatic format, some of the detail and complexity involved is sacrificed.

By way of explanation, accountability circuits in Institutional Ethnography are a subtype of institutional circuits. “Institutional circuits are recognisable and traceable sequences of institutional action in which work is done to produce texts that select from actualities to build textual representations fitting an authoritative or ‘boss’ text (law, policy, managerial objectives, frames of discourse, etc.) in such a way that an institutional course of action can follow” (Griffith & Smith, 2014, p.12). Put more simply, Grace et al. (2014, p.253) explain that as a subtype of institutional circuits, an accountability circuit “is a form of coordination that brings people’s front-line work into alignment with institutional imperatives through the activation of texts”. Stanley (2018, p.51) explains how they “make the activities involved accountable by turning ‘real activities’ into stripped-down institutional categories” depending on a hierarchy of power; “Higher-order texts regulate and standardise texts that enter directly into the organisation of work in multiple local settings” (Smith, 2006, p.79). So, these higher order texts, also called regulatory or boss texts in IE (previously alluded to), organise the institutional categories. Accountability circuits in IE have often been used to show how NPM is introduced into front line organisations, such as in health care, where objectives are set for workers in a way that prefigures how they will later be monitored.

Both institutional and accountability circuits involve what Smith (2006) terms ‘intertextual hierarchy’. This is where boss texts regulate the work of those on the front line which is achieved through activation of subordinate or local texts. Front line workers may not be aware of the boss texts or may not see them as what organises their work on-the-ground. A circular process ensues in the accountability circuit, whereby the subordinate text, recognisable to those on the frontline and produced from the boss text is activated and the work is then circled back to the boss text, to ensure that the boss text’s function is fulfilled. The diagram depicts the accountability circuit that is the focus of this article.

Looking at this accountability circuit, starting with front line work, as IE mandates, the work of standardisation is carried out by the local OSCE team, responsible for running the assessment. This standardising work is what the wider OSCE community see and orientate to. The OSCE team make their work visible to the regulatory body, as their defensibility work; the work processes involved in running the assessment. This work of standardisation, seen outside the medical school as defensibility work is textually represented by the production of a subordinate text, the marksheet. This work in producing the marksheet has been organised by boss texts from the regulatory body; in this research, this is particularly the document “Outcomes for Graduates” from the GMC (2020). The work of the students, SPs, and examiners (the wider OSCE team) orientate to the standardising work through the marksheet; they may not be fully engaged with the boss texts. In addition, to organising on-the-ground work, the boss texts are also used to determine if the defensibility work and documented work processes meet the standards of governance set through the boss texts.

Concentrating specifically on the texts involved (once activated by people in their work), this accountability circuit shows both the dominance of the texts involved in the process (at different levels of the hierarchy, boss versus subordinate) and how the defensibility work, textually represented as the marksheet, is dictated by, and deemed to fit with the regulations. The people involved and their work is being organised by the ideological concept that you can objectively decide who is competent to be a doctor through proving that the assessment categorically meets regulatory standards. The boss texts, activated through the work of those on-the-ground, are used as a framework to map work processes in order to assess the assessments in individual medical schools. Through the subordinate marksheet, the defensibility work to decide which students are competent has been made accountable; once activated, the marksheet reduces the actualities of training medical students into stripped-down institutional categories, marks on a checklist. They are “writing the actual into the technological standardisation that is responsive to the governing frame(s) of the boss texts(s)” (Griffith & Smith, 2014, p.18).

This accountability circuit shows what counts institutionally and for whom; a quantitative based assessment which can be ‘objectively’ deemed to meet ‘quality’ standards through meeting the institutionally mandated regulatory frameworks and satisfying the accountability agenda. In a study previously referenced, Bearman et al. (2021) discuss how they consider the blueprint to be a “key coordinating artefact” of OSCEs. Our work resonates with this suggesting both the blueprint and marksheet as local coordinators, (though only when activated by people carrying out their work) under the organisation of boss texts.

Depicting the sequences of text-mediated work in this way helps us understand the power held in the activation of such texts, strengthening the control that the regulatory body holds over the process. This similarly reflects Bearman et al.’s assertion of increasing cycles of bureaucracy in OSCEs, “This emphasis on bureaucratic process appears associated with an overly reductive underlying set of values” (Bearman et al., 2021, p.648). We bring this argument forward in relation to accountability as the driving force. The untouchable nature of accountability in turn validates the accountability circuit itself rather than the ‘product’ of the circuit—in this case the individual medical student and their practices.

### ‘Accountability-centred practices’ in a time of new public management

In medicine, as in other health professions, there is an oft-cited drive to instil the ethos of person-centred care, often explicitly, in our learners. Our concern is that current discursive practices with a focus on standardisation and defensibility in order to be institutionally accountable, will inadvertently ‘produce’ and validate practices which are accountability-centred rather than being centred on the patient and their social context. Gormley et al. (2016, p.1238) suggested that “OSCEs have potential to promote examiner-centred care”. We seek to extend this, and borrowing some IE phraseology, we suggest that the accountability circuit shows focus towards accountability-centred practices, referring to all endeavours by these future clinicians, not limited to clinical practice. This concept of ‘accountability-centred’ echoes the findings of Nicola Water’s IE thesis on the work of wound care by nurses, where she argues, “far from being person-centred, as the clinically-controlled evidence currently defining wound care is taken up by decision makers and used to organise nurses’ and patients’ work processes, what actually happens is institution centred care” (Waters, 2016, p.237). The Clinical Academics explained how they hoped their assessment would reward students who spent time on the wards with patients and examine what would be needed for the students to start work as doctors six months later. However, we argue that the knowledge of the clinical working environment held by these Clinical Academics has been subsumed and their autonomy in setting the assessment they want constrained by the need to make their work defensible. Bearman et al. advocate for an increasing place for the “professional judgement” of those on-the-ground, in deciding how to coordinate OSCE work as opposed to standardise, even down to the change of language used (Bearman et al., 2021, p.649). We argue here how such professional and experiential judgment of those on-the-ground is not accounted for. Taking this a step further, this resonates with the findings of Townsend’s IE explication of the work of Occupational Therapists in their attempts to advocate for client-centred practice, whereby they face professional tensions as they work at cross-purposes with the prevailing hierarchical management structure, subordinating their attempts. “Tension arises in holding a vision of client quality of life and empowerment while also attempting to meet accountability criteria that favor efficient, acute, medical treatments with individuals while *located bodily in the same time and space*." (Townsend et al., 2003, p.24). In our study, the clinical judgement of those involved in planning for and examining in OSCEs is subsumed by accountability work. Whilst we have used the concepts of ‘examiner-centred care’ and ‘institution centred care’ to build on, we recognise that assessment sits outside direct patient care and have stopped short of using the phrase accountability-centred care; any direct impact of OSCEs on the care provided sits outside the scope of this study. However, high-stakes OSCEs are often the clinical gateway determining which students can gain licensure to be practitioners and within the truism that ‘assessment drives learning’, the discourses that students orientate to within assessment are highly influential. Based on our findings, we are concerned that what is actually ‘high-stakes’ in high-stakes assessments, is that they have become so focused on producing a standardised assessment defensible on many levels, and that, above all, meet the textually represented and textually mediated measures mandated by the regulatory body, that clinical experience is entirely unaccounted for. These observations raise many questions. Does the need for doctors that are above all demonstrably ‘safe’ override other everyday attributes in future doctors that might be desirable for and by patients? Does the accountability of medical schools to produce institutionally ‘competent’ doctors displace and subjugate a person-centred ethos from the newly qualified doctors it ‘produces?’ Does this accountability circuit, placing medical education under new public management ideals disconnect these soon to be doctors from the actualities of the patients that they will encounter? Is accountability above all, ‘what counts?’ And if so, where might patients sit in such an accountability circuit?

Regulatory bodies, through governance of the work processes in medical schools proposing students for medical licence, aim to keep patients ‘safe’, and understandably so. However, with the regulatory body’s primary focus on patient-safety, mandated through accountability, the genuine patient voice which is unstandardised and unstandardisable is not visible at any point in the accountability circuit (noting that *patient voice* itself is not a single entity but instead a myriad of different experiences whether these are frequent or infrequent health system users or even patient representation bodies). The actualities of real patients are displaced, and necessarily so, at least based on the socially organised work processes that determine what counts in accountability. Regulatory bodies, whilst seeing their work as to advocate for the safety of patients, may inadvertently subjugate patients in the process of producing doctors which are above all things, accountable to them. Regulatory bodies may consider their own accountability as an organisation to lie with the public and patients broadly, yet their push for the patient safety discourse above all things displaces patients and their lived actualities from the centre of medical training, promoting accountability-centred practices and practitioners. As Smith (2008, p.26) states, “Though the new public management may have intended to increase the accountability of government to citizens, the circularity of its textual realities means that its management is effectively insulated from the actualities of people’s everyday lives, doings, and work.” Through accountability, new public management, and promotion of the discourse of patient safety, the work of regulatory bodies may have become increasingly distant from the patients that they try to advocate for; the patients that will be cared for in future by the students who receive approval that they are ‘safe’ by the regulatory body as a result of the work processes of their medical school. This displacement of patients resonates with the work on front line paramedics, where Corman concluded that “narrow conceptions of efficiency and accountability are infiltrating the work setting of paramedics and displacing both patients and practitioners as knowing subjects, with problematic and unforeseen consequences” (Corman, 2017, p.15).

### Limitations

Four of the five authors are involved in OSCEs as station designers, examiners and in delivery of OSCE examiner training which will have influenced data collection, analysis, and critique.

This research took place in one undergraduate medical school in the UK and specifically looked at Finals OSCE assessments; work processes in individual medical schools, their choice of assessment method and regulatory bodies differ throughout out the world. However, in IE, the analytical interest lies with how on-the-ground work is organised from a higher-level through texts. Therefore, whilst the specifics of these texts and work processes will vary throughout the world, their influence, which aims to standardise assessment towards an overall focus on accountability should be transferable, towards accountability-centred practices across many geographical and regulatory landscapes.

## Conclusion

In this article, we detailed the work of standardisation in high-stakes assessment and the defensibility work of those involved for their assessments to be ‘passed’ and their graduates deemed competent. This work was traced back to the overriding emphasis on accountability, under the governance of the regulatory body. We mapped this as an accountability circuit, displaying the organising role that texts at various levels of the hierarchy play and highlighting the role of new public management sensibilities in high-stakes clinical assessment. We postulate that these institutional processes displace and distance the actualities of patients and their contexts, mandating a shift towards accountability-centred practices, to the detriment of a person-centred ethos. The paradox is that despite the regulatory body’s aim to advocate for patient safety, as illustrated in the accountability circuit, and described in the analysis above, the social organisation of OSCEs displaces patients from the work processes by which student doctors are granted licensure and start to work with patients. We caution the loss of focus on patients as new public management ideology takes an increasing hold on the education of future health professionals.
